# Major determinant factors of pediatric COVID-19 severity; a single center study

**DOI:** 10.1186/s43054-023-00161-2

**Published:** 2023-04-07

**Authors:** Heba A. Ali

**Affiliations:** Department of Pediatrics, Pulmonology Division, Ain Shams University Children’s Hospital, Faculty of Medicine, Cairo, Egypt

**Keywords:** COVID-19, Clinical characteristics, Egyptian children, Radiological features, Severe

## Abstract

**Background:**

According to several recently published studies, pediatric Corona virus infection is mostly mild. However, a severe COVID-19 illness could occur in children, resulting in grave outcomes. Unfortunately, the data regarding the major determinants of disease progression in the pediatric population is still limited. Here, we aimed to identify the most significant risk factors associated with severe COVID-19 infection in children to predict the patients at elevated risk for serious illness.

**Results:**

This single-center, retrospective study enrolled eighty hospitalized children and adolescents under the age of 18 years with coronavirus type 2 infections, who were divided according to the level of clinical severity into severe and non-severe groups. Epidemiological data, clinical features, radiological findings, laboratory test results, and disease outcomes of the studied patients were collected and analyzed to demonstrate their relation to disease severity. Patients with severe illness tend to have more respiratory symptoms (97.8% vs. 79.4%, *p* = 0.007), cardiac affection (23 (50.0%) vs. 5 (14.7%), *p* = 0.001, and neurological involvement (13 (28.1%) vs. 1 (2.9%), *p* = 0.003). Furthermore, abnormal radiological findings and higher radiological scores were significantly more common among patients with severe disease compared to non-severe cases (*p* = 0.037, 0.013). In multivariable analysis, clinical scoring, abnormal coagulation function, and ICU admission were the most significant parameters for forecasting severe illness.

**Conclusions:**

We identified the most remarkable parameters involved in the progression of severe disease in Egyptian children with COVID-19 infection, which may be implemented in anticipation of susceptible children for earlier prompt management and a better prognosis.

**Supplementary Information:**

The online version contains supplementary material available at 10.1186/s43054-023-00161-2.

## Background

Coronavirus Disease 2019 (COVID-19), caused by severe acute respiratory syndrome coronavirus 2 (SARS-CoV-2), was first identified in Wuhan, China, in December 2019. It has rapidly spread across the world. The World Health Organization (WHO) had officially declared the disease a pandemic and a public health emergency [1]. Egypt reported the first cases of COVID-19 on February 14, 2020, with an initial daily increase in the number of confirmed cases. In Egypt, from January 3, 2020, to February 17, 2023, there have been 515,698 confirmed cases of COVID-19, with 24,809 deaths, reported to WHO [2].

The severity of symptoms among patients infected with COVID-19 varies considerably, from asymptomatic infection to critical illness with serious complications [3, 4]. According to international pediatric studies, children have lower rates of severe COVID-19 infection than adults [5, 6], which may be due to differences in immune system responses [7], and infected children typically have a good prognosis [8]. However, the higher prevalence of coronavirus 2 in Egypt increases the probability of having a severe pediatric illness. Although some researchers have suggested that there are several factors that might be responsible for the severity of COVID-19 infection among adults [9, 10], the potential factors responsible for severe cases in pediatrics have not been clearly identified.

In the medical literature, reports regarding the clinical features of children with COVID-19 have distinct results that may be peculiar to each population [11, 12]. Addressing the characteristics of the children with severe COVID-19 infection could assist healthcare providers in proper medical decision-making to improve the management of these vulnerable children and develop predictive tools to identify children at risk for clinical deterioration, in particular in countries with limited resources.

Therefore, this study was conducted to compare demographics, clinical characteristics, radiological findings, laboratory parameters, and treatment options among children and adolescents infected with severe and non-severe COVID-19 infection, to explore the potential risk factors associated with disease severity, and to examine the hypothesis that some potential risk factors may be attributed to severe COVID-19 illness in children. If this hypothesis is settled, we can further establish a risk profile model to facilitate categorization of the disease severity in pediatric patients.

## Methods

### Study settings

We carried out a retrospective study, including 80 COVID-19 confirmed cases, during the period from April 2021 through the end of June 2022. The study sample included all the consecutive patients admitted to Ain Shams University Children’s Hospital during the study period. The hospital is a tertiary care pediatric hospital, and it is one of the major referral hospitals in Cairo, Egypt. This hospital was designated as a pediatric COVID-19 center during the pandemic, and children with suspected or confirmed COVID-19 infection were transferred there. The treatment was administered according to the Egyptian National Guidelines for Clinical Management and Treatment of COVID-19 [13].

### Study participants

The study enrolled hospitalized children under the age of 18 with confirmed COVID-19 infection using real-time reverse transcriptase-polymerase chain reaction (RT-PCR) [14], who were admitted to the hospital during the study period, including patients admitted for other illnesses such as malignancy, diabetic ketoacidosis, or polytrauma and developed COVID infection during their hospital stay. No exclusion criteria were applied. The studied patients were classified into severe and non-severe cases. A patient was diagnosed with a severe disease when any of the following criteria were met: 1) Adolescent COVID-19 patients with clinical signs of pneumonia (i.e., fever, cough, dyspnea, fast breathing) plus one of the following: respiratory rate > 30 breaths/minute, severe respiratory distress, or oxygen saturation < 90% on room air. 2) COVID-19 children and infants with clinical signs of pneumonia (i.e., cough or difficulty in breathing) plus at least one of the following: central cyanosis or oxygen saturation < 90%, severe respiratory distress (e.g., tachypnea according to age group, grunting, very severe chest indrawing), inability to breastfeed or drink, lethargy, unconsciousness, or convulsions. 3) respiratory failure requiring mechanical ventilation; 4) shock; or 5) other organ failure requiring intensive care [15]. A patient was discharged when all four criteria were met, including: 1) being afebrile for more than 3 days; 2) having respiratory symptoms that have remarkably improved; 3) improvement in the chest CT radiographic findings; and 4) having two consecutive negative RT-PCR for viral COVID-19 nucleic acid at least 24 h apart [15, 16].

### Data collection

Data were collected from patients’ medical records and rechecked with the patients' caregivers by the study physician. The collected data included basic demographic information including age, sex, residence, and socioeconomic status using the El-Gilani score [17], exposure to passive smoking, history of any chronic illness), history of exposure to a confirmed case of COVID or household contact among the family members using laboratory confirmation of SARS-CoV-2, and history of travel to endemic countries in the last two weeks before the presentation. time of disease onset, duration of symptoms before presentation, duration of hospital stays, and the duration of illness, which was defined as time from the onset of the disease till outcome. In addition, the presenting symptoms and signs compatible with COVID infection, as reported in the literature [18, 19], were collected on admission, including fever (temperature ≥ 38 °C), cough, dyspnea, bony aches, sore throat, loss of taste or smell, vomiting, diarrhea, or abdominal pain, extreme fatigue and/or irritability, respiratory distress, which was identified by persistent tachypnea and/or use of accessory muscles documented during physical examination, symptoms suggestive of multi-system inflammatory syndrome in children (MIS-C), such as non-allergic conjunctivitis, skin rash, cracked lips, and changes in hands and feet, MIS-C was defined as a clinically severe illness requiring hospitalization with fever, elevated inflammatory markers, and multisystem organ dysfunction in the setting of current, recent, proven, or probable COVID-19 infection and in the absence of an alternative likely explanation [20]. In addition to the medications prescribed, other comorbidities such as chronic respiratory diseases, immunosuppression, or malignancy were documented. Disease severity and clinical course during admission were recorded.

Moreover, laboratory test results were recorded and analyzed, including complete blood cell counts along with a white blood cell count, with special focus on the presence of lymphopenia, which was defined as an absolute lymphocyte count (ALC) of less than 4500/ μL in infants under the age of 8 months or less than 1500/ μL in patients 8 months and older [21], neutrophil/lymphocyte ratio, platelet count, hemoglobin, the international normalized ratio, liver and renal function tests, and inflammatory markers such as C-reactive protein (CRP), erythrocyte sedimentation rate (ESR), lactate dehydrogenase (LDH), serum ferritin, fibrinogen, and D-dimer levels. An electrocardiogram and an echocardiogram performed for patients with suspected cardiac involvement were also recorded. The results of imaging studies, including chest X-rays (CXR) and computed tomography (CT) of the chest, were interpreted in accordance with the recommendations of the Radiological Society of North America (RSNA) [22], the radiological findings were classified as normal or abnormal, including a detailed description of radiological abnormalities enclosing the presence of bilateral ground glass opacities, diffuse or focal consolidation, pleural effusion, numbers of lobes affected, and the largest opacity size (mm). Finally, the need for intensive care unit (ICU) admission and/or mechanical ventilation and patient outcomes, either discharged or dead, were also recorded.

### Severity assessment

All the studied patients were evaluated for the risk of severe illness using the following severity scores:

1. CXR score: It is a radiological score aimed at evaluating COVID-19 manifestations in the lung. It considered the presence of interstitial and/or alveolar abnormalities, as described by Borghesi et al [23]. Mild and prevalent interstitial disease in different lung fields corresponded to a CXR score of 0–3, while multiple alveolar consolidations, suggesting bilateral or multi-lobar alveolar disease, corresponded to a CXR score ≥ 6.
2. Chest CT Score: In this score, from the apex to the bottom, the lung was divided into five levels: the suprasternal notch, the aortic arch, the tracheal carina, the intermediate bronchus, and the apex of the diaphragm. The left and right lungs were scored separately, and each of the 5 lung zones in each patient was assigned a score according to the distribution of affected parenchyma as previously described [24]. The chest CT density was also graded (0, normal attenuation; 1, frosted glass density; 2, ground-glass attenuation; and 3, consolidation). Then the lung parenchyma score was multiplied by the square of the CT density score and points from all zones and added for a final total cumulative score that ranged from 0 to 900.
3. CT severity score (CT-SS): The CT-SS is used to describe ground-glass opacity, interstitial opacity, and air trapping in SARS infection [25]. According to the anatomic structure, the 18 segments of both lungs were divided into 20 regions, in which the posterior apical segment of the left upper lobe was subdivided into apical and posterior segmental regions, whereas the anteromedial basal segment of the left lower lobe was subdivided into anterior and basal segmental regions. The lung opacities in all 20 lung regions were evaluated on chest CT images using scores of 0, 1, and 2 if parenchymal opacification involved 0%, less than 50%, or equal to or more than 50% of each region, respectively. The CT-SS was defined as the sum of the individual scores in the 20 lung segment regions, which may range from 0 to 40 points.
4. COVID-19 severity index: This score is used to assess COVID-19 severity using a set of selected clinical and laboratory variables [26]. The variables included age, male sex, respiratory rate, oxygen saturation, heart failure, diabetes, systolic blood pressure, temperature, pulse, D-dimer, dyspnea, lymphocytes, and platelet count. Patients were divided into four risk categories based on their score: low risk (0–2), moderate risk (3–5), high risk (6–7), Critical 8 or more (Supplementary Table 3).

### Sample size calculation

The consecutive non-probability sample size technique has been used. Using the PASS11 program for sample size calculation and, based on a previous study [27], for the differentiation between the two study groups (severe versus non-severe), A sample size of at least 34 patients in each group achieved a study power of 80 percent to detect significance for the comparisons between the two groups, assuming an alpha error of 0.05 and a beta error of 0.2. The estimated study population frequency of the outcome factor is 61.1%, with a margin of error of ± 5. The design effect is 1 with a 95% confidence level.

### Statistical analysis

All statistical calculations were done using IBM SPSS version 23. For quantitative data, mean standard deviation (SD), medians, and interquartile range (IQR) were used, while for qualitative data, frequencies (number of cases) and percentages were used. P values less than 0.05 were considered statistically significant, and P values less than 0.01 were considered highly significant. The kappa statistic was used to test the inter-rater reliability of the severity scores. A bivariate analysis was carried out comparing patient characteristics, radiological findings, comorbidities, and laboratory parameters between the severe and non-severe groups. This was conducted using nonparametric tests (the Mann–Whitney U test) or parametric tests (the Student's t-test) for continuous variables, as appropriate.

## Results

### Study Population

Between April 2021 and June 2022, 80 unique patients, whose ages ranged from 2 months to 16 years, who tested positive for SARS-CoV-2 infection were included in the current study. 34 (42.5%) were classified as non-severe, while 46 (57.5%) were classified as severe. The median ages of the studied patients were 5.5 (IQR: 2.5–10) and 6 (IQR: 1.5–10) years among the non-severe and severe groups, respectively. The majority of patients (70.6% and 60.9%, respectively) were in the scholar and preschooler age groups in both the non-severe and severe groups. The proportion of females was significantly higher among severe group 28 (60.9%) versus non-severe group 13 (38.2%) (*p* = 0.018). In addition, low socioeconomic status was significantly observed among severe group 18 (39.1%) compared to non-severe group 9 (26.5%; *p* = 0.049). The median time from the development of symptoms till admission was 2 days (IQR, 1–3) among the non-severe group and 2 days (IQR, 1–2) among the severe group. 62 of these 80 patients (77.5%) acquired SARS-CoV-2 in the community, while 10 (29.4%) and 8 (17.4%) patients developed health care-associated infections among the non-severe and severe groups, respectively. A history of direct exposure to a confirmed case of COVID-19 infection was reported among 18 (39.1%) of the severe group and 7 (20.6%) of the non-severe group. Comorbidities were observed among 21 (45.6%), 15 (44.1%) of severe, and non-severe groups sequentially (*p* = 0.890). Baseline characteristics of the recruited patients are presented by degree of severity in Table 1.

**Table 1:** Demographics and basic characteristics among the studied patient groups

Variables	Non severe No. = 34	Severe No. = 46	P-value
Age (in years) Median (IQR)	5.5 (2.5–10)	6 (1.5–10)	0.578
Range	0.25 – 16	0.17 – 15	
Age categories			
Infant (4 weeks – 1 year)	5 (14.7%)	10 (21.7%)	0.635
Toddler (12 m-24 m)	3 (8.8%)	5 (10.9%)	
Preschooler (2–5 years)	10 (29.4%)	7 (15.2%)	
Scholar (6–13 years)	14 (41.2%)	21 (45.7%)	
Adolescent (14–17)	2 (5.9%)	3 (6.5%)	
Gender			
Female	13 (38.2%)	28 (60.9%)	0.018*
Male	21 (61.8%)	18 (39.1%)	
Residence			
Urban	23 (67.6%)	34 (73.9%)	0.540
Rural	11 (32.4%)	12 (26.1%)	
Socioeconomic status			
Low	9(26.5%)	18 (39.1%)	0.049*
Middle	22 (64.7%)	28 (60.9%)	
High	3 (8.8%)	0 (0.0%)	
Weight(kg), Median (IQR)	20.5 (12–30)	17 (10–30)	0.465
Range	4 – 46	3 – 50	
Height(cm), Mean ± SD	111.29 ± 27.34	107.15 ± 31.21	0.538
Range	61 – 166	60 – 160	
Body mass index (kg/m2), mean ± SD	16.64 ± 4.16	16.10 ± 4.51	0.588
Range	8.2 – 25	6.2 – 27.8	
Time of disease onset (days)			
Before hospitalization, median (IQR)	2 (1–3)	2 (1–2)	0.386
After hospitalization, median (IQR)	2 (2–4)	4 (2–6)	0.546
Duration of the disease(days), median	12 (9–17)	12 (10–17)	0.629
Range	4 – 27	5 – 30	
Duration of hospitalization(days)	10 (7–14)	12 (7–16)	0.439
Range	3 – 25	5 – 28	
History of exposure to COVID patient	7 (20.6%)	18 (39.1%)	0.077
Comorbid medical conditions (%)	15 (44.1%)	21 (45.6%)	0.890

P*; Significant

### Clinical presentations

As shown in Table 2, the reported signs and symptoms of the study population were compared according to the level of severity. For the entire study population, fever, wheezes, and dyspnea were the most common symptoms upon admission. The patients with severe disease tended to have a higher number of upper respiratory tract symptoms: 45 (97.8%), fever 45 (97.8%), wheezes 34 (73.9%), dyspnea 32 (69.6%), and respiratory distress 32 (69.6%) (P < 0.001) compared with those without severe disease: 27 (79.4%), 28 (82.4%), 16 (47.1%), and 15 (44.1%) (*p* = 0.007, 0.015, 0.014, 0.022, 0.022) The percentage of patients with cardiac manifestations (including arrhythmia, myocarditis, and heart failure) and hypotension, was significantly higher among the severe group (*P* = 0.001, 0.002). Atypical presentations including neurological symptoms, seizures or conjunctivitis were significantly observed among severe group than non-severe group (*p* = 0.003, 0.017, 0.029). Moreover, the proportion of patients with COVID complications, and MIS-C were significantly more prevalent among severe group than non-severe groups (*p* = 0.002).

**Table 2:** Clinical characteristics among the studied patients’ groups

Variables	Non severe No. = 34	Severe No. = 46	*P*-value
Upper respiratory tract symptoms	27 (79.4%)	45 (97.8%)	0.007**
Lower respiratory tract symptoms	15 (44.1%)	32 (69.6%)	0.022
Fever	28 (82.4%)	45 (97.8%)	0.015*
Grades of fever			
Normal	7 (20.6%)	1 (2.2%)	0.027*
Mild grade	8 (23.5%)	15 (32.6%)	
Moderate grade	9 (26.5%)	20 (43.5%)	
High grade	10 (29.4%)	10 (21.7%)	
Cough	13 (38.2%)	16 (34.8%)	0.751
Type of cough			
Dry	11 (32.4%)	14 (30.4%)	0.927
Productive	2 (5.9%)	2 (4.3%)	
Sore throat	11 (32.4%)	15 (32.6%)	0.981
Wheezes	16 (47.1%)	34 (73.9%)	0.014*
Dyspnea	15 (44.1%)	32 (69.6%)	0.022*
Respiratory distress	15 (44.1%)	32 (69.6%)	0.022*
Gastrointestinal tract symptoms	14 (41.2%)	29 (63.0%)	0.052
Conjunctivitis	0 (0.0%)	6 (13.0%)	0.029*
Neurological symptoms	1 (2.9%)	13 (28.3%)	0.003**
Convulsions	0 (0.0%)	7 (15.2%)	0.017*
Cardiac manifestations	5 (14.7%)	23 (50.0%)	0.001**
Hypotension	3 (8.8%)	18 (39.1%)	0.002**
Complications of covid-19			
None	22 (64.7%)	14 (30.4%)	0.002**
MIS-C	2 (5.9%)	16 (34.8%)	0.002**
Pneumonia	7 (20.6%)	7 (15.2%)	0.532
Septic shock	2 (5.9%)	6 (13.0%)	0.291
Encephalitis	0 (0.0%)	2 (4.3%)	0.218
Pleural effusion	1 (2.9%)	4 (8.7%)	0.387

*MIS-C* Multisystem inflammatory syndrome in children

P*; Significant

P**; Highly significant

### Radiographic findings

CXR and chest CT were performed for all the studied patients. Normal radiological findings were significantly higher in the non-severe group (19) (55.9%) than in the severe group (15) (32.6%) (*P* = 0.037). Abnormal findings, including consolidation, were significantly more common in group 20 (43.5%) than in group 3 (8.8%) (*p* = 0.001). Typical radiographic findings specific to COVID infection were reported in 7 (15.2%) of the severe group versus 0 (0.0%) in the non-severe group (*p* = 0.053). The patients in the severe group are more likely to have a higher frequency of lobe involvement 3 (0–4) and a larger opacity size (mm) 4 (0–5) than those in the non-severe group 0 (0–3), 0 (0–4) (*P* = 0.024). Regarding radiological severity, the median CXR score, chest CT severity score (CT-SS), chest CT score, and COVID-19 severity index were significantly higher among the severe group than the non-severe group (*p* = 0.019, 0.013, 0.013, 0.001). The radiological features were illustrated in Table 3 and Supplementary Fig. 1.

**Table 3:** Radiological findings among the studied patients’ groups

Variables	Non severe No. = 34	Severe No. = 46	P-value
Computed tomography of chest findings			
Negative	19 (55.9%)	15 (32.6%)	0.037*
Ground glass opacities (GGOs)	4 (11.8%)	7 (15.2%)	0.657
Consolidation	3 (8.8%)	20 (43.5.%)	0.001**
Ground glass opacities (GGOs) & consolidation	0 (0.0%)	2 (4.3%)	0.218
Bronchiectasis changes	1 (2.9%)	0 (0.0%)	0.242
RSNA expert consensus statement			
Negative	19 (55.9%)	15(32.6%)	0.053
Indeterminate	10 (29.4%)	13 (28.3%)	
Typical	0 (0.0%)	7 (15.2%)	
Atypical	5 (14.7%)	10 (21.7%)	
Frequency of lobe involvement, median (IQR)	0 (0–3)	3 (0–4)	0.024*
Range	0 – 6	0 – 6	
Largest opacity size (mm), median (IQR)	0 (0–4)	4 (0–5)	0.024*
CXR score, median (IQR)	1 (0–3)	3.5 (0–4)	0.019*
Range	0 – 6	0 – 6	
Chest CT severity score (CT-SS), median (IQR)	0 (0–12)	12 (0–24)	0.013*
Range	0 – 32	0 – 32	
Chest CT score, median (IQR)	0 (0 – 160)	155 (0 – 360)	0.013*
Range	0 – 540	0 – 720	
COVID-19 Severity Index, median (IQR)	5 (3–7)	12.5 (8–15)	0.001**
Range	1 – 14	4 – 18	
Low risk	8 (23.5%)	0 (0.0%)	
Moderate risk	13 (38.2%)	3 (6.5%)	0.001**
High risk	9 (26.5%)	6 (13.0%)	
Critical	4 (11.8%)	37 (80.4%)	

P*; Significant

P**; Highly significant

### Laboratory data among the studied groups

On admission, the white blood cell counts were generally normal (median 8.95 (6.8–16.6) versus 12.35 (7.6–18.2) among the non-severe and severe groups, respectively. In addition, lymphopenia at admission did not differ significantly in patients with 18, (39.1%) and without severe disease (12, (35.3%). Moreover, patients with severe illness showed a significantly increased neutrophil/lymphocyte ratio, 3.51 (1.23–4.69) compared to those with non-severe disease 1.38 (1 – 3.72) (*p* = 0.039). Serum ferritin 592.65 (314–1150) and partial thromboplastin time 37.5 (34.3–43.3) were significantly higher at admission for patients with severe disease versus patients with non-severe disease (356 (172–495) and 33.35 (28.25–40.6), respectively (*p* = 0.013, 0.036). Table 4 displays the laboratory results for both patient groups.

**Table 4:** Laboratory investigations among the studied patient groups

Variables	Non severe No. = 34	Severe No. = 46	*P*-value
White blood cells (10^3/ul), median (IQR)	8.95 (6.8–16.6)	12.35 (7.6–18.2)	0.259
Range	2.1 – 31.6	0.5 – 34.3	
Normal leucocytic count	16 (47.1%)	23 (50.0%)	0.702
Leucopenia	5 (14.7%)	4 (8.7%)	
Leukocytosis	13 (38.2%)	19 (41.3%)	
Neutrophils (10^3/ul), median (IQR)	6.2 (2.52–10.5)	7.85 (4.6–12.4)	0.130
Range	0.4 – 24.9	0.2 – 26.2	
Normal neutrophil count	17 (50.0%)	23 (50.0%)	0.430
Neutropenia	4 (11.8%)	2 (4.3%)	
Neutrophilia	13 (38.2%)	21 (45.7%)	
Lymphocytes (10^3/ul), median (IQR)	3.07 (1.4–4.7)	2.6 (1.6–4.4)	0.439
Range	0.9 – 24.9	0.2 – 12.2	
Normal lymphocyte count	16 (47.1%)	23 (50.0%)	0.682
Lymphopenia	12 (35.3%)	18 (39.1%)	
Lymphocytosis	6 (17.6%)	5 (10.9%)	
Neutrophil/lymphocyte ratio, mean ± SD	1.38 (1 – 3.72)	3.51 (1.23 – 4.69)	0.039*
Hemoglobin(g/dl), (mean ± SD)	10.12 ± 1.76	10.18 ± 2.07	0.879
Platelets (10^3/ul), median (IQR)	391 (190–546)	348 (187–475)	0.523
Serum ferritin (ng/ml), median (IQR)	356 (172 – 495)	592.65 (314 – 1150)	0.013*
Covid19 antibodies, median (IQR) Range	9.2 (0.1–39.2)	9.95 (0.1–22)	0.914
	0.1 – 60.1	0 – 54.4	
D.- dimer (mcg/mL FEU), median (IQR)	1.44 (0.52 – 3.07)	1.94 (0.8 – 3.92)	0.192
Range	0.27 – 20	0.28 – 20	
Normal D—dimer level	10 (29.4%)	8 (17.4%)	0.203
High D- dimer level	24 (70.6%)	38 (82.6%)	
Fibrinogen(g/l), mean ± SD	3.70 ± 1.15	3.78 ± 2.13	0.959
Total Creatine kinase (IU/L), median (IQR)	52 (21 – 116)	62 (24 – 103)	0.960
Troponin (ng/ml), median (IQR)	0.005 (0.003 – 0.064)	0.021 (0.002 – 0.066)	0.650
C-Reactive Protein (mg/L), median (IQR)	39.55 (7.4 – 143.4)	50.7 (6.8 – 219.8)	0.589
Aspartate aminotransferase, median (IQR)	30.5 (22–50)	29.5 (21–52)	0.919
Alanine transaminase, median (IQR)	16 (12–20)	19 (13–38)	0.250
Lactate dehydrogenase (IU/L), median (IQR)	338.5 (291 – 541)	369.5 (314 – 492)	0.789
Albumin (g/dl), mean ± SD Range	3.17 ± 1.14	3.00 ± 0.97	0.481
Partial thromboplastin time (sec), median (IQR)	33.35 (28.25–40.6)	37.5 (34.3–43.3)	0.036*
Prothrombin time (sec), mean ± SD	16.13 ± 3.62	16.44 ± 4.47	0.792

P*; Significant

P**; Highly significant

### Drug therapy and disease outcomes

Antibiotic therapy was the most commonly used drug overall (100%). Antiviral drugs were the second most common type of therapy administered for non-severe cases (15; 44.1%) and severe cases (18; (39.1%) respectively. 38 (82.6%) of the patients in the severe group received anticoagulant therapy, compared to 17 (50%) in the non-severe group (*p* = 0.054). A higher proportion of patients with severe conditions (primarily those with MIS-C) were treated with IVIG than those who were not (33 (71.7%) versus 10 (29.4%) (*P* = 0.001). Significantly more patients with severe disease were admitted to the intensive care unit (ICU): 42 (91.3%) versus 0 (0.0%), and received mechanical ventilation: 26 (56.5%) versus 0 (0.0%) in the non-severe group (*p* = 0.001 for all). In terms of patient outcomes, death was more significantly reported among severe patients: 12 (26.1%) versus 0 (0.0%) in the non-severe group (*p* = 0.001). Treatment measures and study outcomes for the studied patient groups are shown in Supplementary Table 1.

### Risk factors for severe disease

We identified more than 19 risk factors in four categories (demographic characteristics, clinical data, radiological findings, and laboratory results), which were found to be significantly associated with disease severity relative to non-severe illness (specifically female sex, low socioeconomic status, respiratory symptoms, the presence of MIS-C, neurological affection, involvement of more than 3 lung lobes, CXR score > 3, chest CT score > 160, chest CT severity score > 9, COVID-19 severity index > 7, neutrophil/lymphocyte ratio > 1.54, partial thromboplastin time (sec) > 35.4, and serum ferritin (ng/ml) > 537.

According to the multivariate logistic regression analysis, the most important determinant factors associated with increased risk of severe illness among the studied patients were clinical manifestations, specifically the lower respiratory symptoms, with an OR (95% CI) of 31.359 (5.251–187.277) and p-value 0.001, followed by prolonged partial thromboplastin time (sec) > 35.4 with an OR (95% CI) of 18.763 (0.696- 505.502) and *p*-value = 0.012.All statistically significant factors in our univariate and multivariate analyses are summarized in Supplementary Table 2.

## Discussion

COVID-19 is currently a major infectious disease, causing substantial morbidity and mortality worldwide [28]. Coronavirus diseases can manifest as mild, moderate, severe, or even fatal respiratory infections [11]. A proper understanding of the leading causes of severe illness may decrease the disease burden and fatality rates through early management to prevent serious complications. Moreover, it reduces unnecessary use of the healthcare system and reserves health care services for high-risk children who are really in need of more intensive care, thus reducing the economic burden during this pandemic.

In this work, we described the epidemiology, clinical characteristics, imaging findings, laboratory results, and disease outcomes of pediatric patients hospitalized with confirmed COVID-19 infection and compared these findings between severe and non-severe patient groups. The current study revealed that clinical severity, an abnormal coagulation profile, and the need for ICU admission were the most significant factors associated with COVID-19 severity among the studied population.

To the best of our knowledge, this is one of the few studies that looked at the characteristics of severe and non-severe children and adolescents infected with SARS-CoV-2 in our community.

In our study, the median age of the studied patients was 6 years (IQR: 2–10). Our findings are in line with a previous meta-analysis [29] including 203 children, which found that the mean age of the studied patients was 5.46 years.

The current study revealed no statistically significant difference between severe and non-severe groups regarding age, which was incongruent with a previous study from China, which reported that infants and younger children are more likely to develop severe clinical manifestations than older children, likely due to an immature immune system [30]. On the other hand, another recent study [31] revealed that infants had lower odds than older children of progressing to severe or critical disease. This difference between the studies may be due to the smaller percentage of infants and toddlers in our study population.

Our results revealed that 18 (22.5%) of the studied patients acquired COVID infection after hospitalization (Table 1), which necessitates the implementation of intensive infection control policies to reduce the risk of potential viral transmission and alleviate the burden on the healthcare system.

This work revealed the predominance of severe disease (57.5%) in comparison to non-severe disease (42.5%) among our pediatric population, which came against several published studies that stated that children have less serious diseases that necessitate hospitalization [12, 18]. Moreover, a meta-analysis [32] based on 52 case reports, including 203 children with COVID-19, revealed that only 15 (7.46%) were diagnosed as severe cases.

The higher percentage of severe cases among our study participants compared to the previous studies raises awareness about the high possibility of severe disease among children, especially in communities with a high prevalence of COVID infection, and the urgent need for prognostic tools for the early detection of high-risk children vulnerable to disease progression.

In the present study, the percentage of females was significantly higher among the severe group (*p* = 0.018), which disagrees with several published studies that showed that males were more likely to have severe disease than females [33–35] which was explained by the differences in infection susceptibility, inflammation, and immune response between females and males [36, 37].

Our study revealed that patients with severe COVID-19 infection had significantly lower socioeconomic status (SES) (*p* = 0.049) (Table 1), which agrees with what has been reported in the literature that communities with a high percentage of lower-income individuals are suffering from a greater rate of COVID-19 infection [38, 39]. In addition, Danielle et al., [40] found that 31% of patients hospitalized with SARS-CoV-2 were of low SES, where low earnings are associated with poor health outcomes and frequent ICU admissions.

The current study found that fever, respiratory symptoms, and gastrointestinal symptoms were the most common symptoms among the studied patients, followed by cardiac manifestations and atypical presentations (Table 2), which agrees with a recent study [19], which revealed that most COVID-19-infected children had fever (80%), upper and lower respiratory tract symptoms (64%), and atypical presentations including seizures or seizure-like activity (6%). Our findings were also consistent with the findings of Lechien et al. [41], lqahtani et al. [28] in adults.

In this study, 43 (53.75%) of the studied patients reported gastrointestinal symptoms, which were slightly higher among the severe group (63.0% versus 41.2%) (*p* = 0.052) (Table 2).

In agreement with our findings, A recent study [40] reported that up to half of the patients reported 1 of the gastrointestinal symptoms, whereas it was reported in less than one-quarter of hospitalized adults with COVID infection [42, 43].

Compared to our study, a previous pediatric study [19] reported that gastrointestinal tract symptoms were observed in only 7 patients (14%) with Corona virus infection.

The high percentage of gastrointestinal symptoms among our patients may be due to high prevalence of associated viral gastroenteritis in our tropical communities, and the predominance of severe cases among the studied population, therefore, their findings were close to adult patients.

Neurological presentation was significantly more common among patients with severe disease (*p* = 0.003) (Table 2), which is in line with a recently published study that suggested that neurological involvement was quite common in COVID-19, especially in severe cases [44]. In addition, neurologic manifestations have been documented in adults with COVID-19 infection [45].

The neurological ailment could be explained by the SARS-CoV-2 virus's ability to infect nerve cells via the olfactory, trans-synaptic, leukocytic, or haematogenic routes [46].

The cardiac involvement, especially MIS-C, was significantly higher among the severe group (*p* = 0.001, *p* = 0.002) (Table 2), which agrees with a recent study conducted by Li B et al., [47], who reported that the incidence of cardiac involvement in COVID-19 infection was nearly 13 times higher in severe patients than non-severe patients. Moreover, a recent adult study [48] stated that MIS-C was reported in combination with manifestations of severe pulmonary COVID-19 as evidenced by the respiratory symptoms along with the extensive pulmonary affection in the CT (CO-RADS 4 and 5).

The cardiac affection may be caused by viral replication, which may cause direct cardiac damage through downregulation of angiotensin-converting enzyme 2 (ACE2), or indirect damage, which may be mediated through the release of cytokines, coagulopathy, insulin resistance, or an immune inability to fight inflammatory pathogens as in MIS-C [49].

We observed that conjunctivitis was significantly seen only among patients with severe illness (*p* = 0.029) (Table 2), which is consistent with a study conducted by Wu et al. [50] who observed that ocular abnormalities occurred more frequently in patients with more severe COVID-19 illness. In addition, a recent study [40] reported that ocular and dermatologic findings were observed in 32% and 39% of MIS-C cases, which were rarely reported in adults.

Our study revealed that the most common radiological abnormalities were consolidation and ground glass opacities (GGOs), followed by mixed GGOs and consolidation in both severe and non-severe groups, which were consistent with previous reports [51]. However, consolidation was significantly higher among the severe group than the non-severe group (*p* = 0.001). In addition, disease severity was significantly associated with higher radiological and clinical severity scores (*p* = 0.019, 0.013, 0.013, 0.001) (Table 3; Supplementary Fig. 1).

Consistent with our report, previous studies declared that patients who had viral pneumonias with CT evident bilateral consolidations, had more severe clinical courses than those who presented with ground glass opacities [52, 53]. These abnormalities may be correlated with severe systemic inflammation and alveolar damage, which indirectly reflect the disease severity in the study participants.

Furthermore, a recent study [54] of 76 pediatric patients with confirmed COVID-19 found a significant relationship between chest CT score and COVID-19 severity, suggesting its use to assess disease severity.

Our results revealed that the proportion of patients in need for ICU admission and mechanical ventilation was significantly higher among the severe cases compared to the non-severe group (*p* = 0.001) (Supplementary Table 1).

Similar to our findings, a previous cohort study [55] revealed that the severe/critical and mortality rates in pediatric COVID-19 cases were relatively high.

The findings of our study are in discordance with other published studies [8, 56] which reported that children have a better prognosis for COVID-19, when compared to adults, which was explained by the difference in the immune system response to viral infection [56, 57].

The difference between our findings and previous studies may be due to recent mutations in the virus strains and the appearance of new variants that may be more aggressive, increasing the susceptibility to severe disease in vulnerable children.

Our study revealed a high percentage of mortality (16.25%), which was predominately reported among the severe group (*p* = 0.001) (Supplementary Table 1). Our findings were remarkably higher than previous publications [28, 40], where the overall mortality rate was only 2%, or 0.44%, all in the severe group. This distinction emphasizes the significance of precise risk assessment of pediatric patients with COVID-19 infection in our community, as well as the need for early aggressive interventions for high-risk children to reduce mortality.

In the present study, patients with severe disease displayed significantly higher inflammatory markers, such as serum ferritin and the neutrophil/lymphocyte ratio, compared to those with non-severe disease (*p* = 0.013 and 0.039, respectively) (Table 4). Significantly increased inflammatory markers suggestive of an overactive immune response and a hyperinflammatory state were mostly seen in patients with severe disease, as described in adults [58].

This was in conformity with previous studies that reported marked elevations of serum ferritin in patients with severe COVID-19 infection [59], which can be explained by the fact that serum ferritin is an acute-phase reactant that rises during the inflammatory process of Corona virus infection.

Our findings are also consistent with a previous study [60], which found that patients infected with COVID-19 who had an elevated neutrophil/lymphocyte ratio were more likely to develop critical illness, and it may be a predictor of disease severity; this was also established in other multiple studies [61–63].

This finding may be explained by the negative effect of COVID-19 infection on T lymphocytes counts [64], in addition to an elevated total leucocytic count, which is mediated by infection-induced proinflammatory cytokines produced during severe illness [65].

The present study revealed that the most significant determinants of disease severity were symptom severity, coagulation dysfunction based on prolonged activated partial thromboplastin time (aPTT), and ICU admission. (P 0.001, 0.012, 0.001), which have been mostly associated with poor prognosis (Supplementary Table 2).

Our results are consistent with a recent meta-analysis of research [66], which found 11 factors to be significantly associated with the risk of severe illness, especially for dyspnea and tachypnea, an abnormal chest X-ray, age, comorbidity, cough, and LDH.

On the same hand, Tan et al. [67] and Bowles et al. [68] reported that patients with severe COVID-19 had mostly mildly prolonged aPTT clotting times. While previous studies [69, 70], reported that the levels of aPTT differed negligibly between severe and mild patients, Our finding may be due to the presence of lupus anticoagulants among COVID-19 patients, as reported by previous publications [68, 71, 72].

On the other hand, a subset of COVID-19 patients can have an abnormally short aPTT [73], which is often related to elevated factor VIII [74], which acts as an acute-phase reactant to a severe COVID-19 induced inflammatory response [75].

In contrast to our findings, a recent study [40] reported that obesity, increasing white blood cell count, younger age, hypoxia, and bilateral infiltrates on chest radiographs at admission were independent predictors of severe disease.

Another pediatric registry [76] conducted in Asia revealed that the risk factors for severe disease in children were age younger than 12 months, presence of comorbidities, and cough at presentation.

On the other hand, a national prospective surveillance study [77] conducted in Canada reported that severe COVID-19 was detected across all age groups and in children with and without comorbid conditions.

This difference between our study and the previous studies may be due to the variability in the age groups, clinical presentations, and methods of severity assessment among the studied populations.

## Study limitations

This study has some limitations. First, the study was performed in a single center, so our findings may not be generalizable to the whole pediatric population with COVID-19 infection. Second, some of the data may have been missed in this retrospective study, so further multicentric longitudinal studies applied to larger numbers of patients should be done to confirm our results. However, it is important to note that the findings in the present study have not been previously reported.

## Conclusions

The percentage of severe illness among children and adolescents with COVID-19 infection was higher in this study than previously reported. In addition, atypical presentations were displayed in our study with a high prevalence of cardiac involvement. The study identified the major determinants of pediatric COVID-19 severity that should be used in clinical practice to identify children at high risk for disease progression and provide prompt management to reduce future complications and mortality while conserving medical resources, particularly in low-income countries.

## Supplementary Information

**Additional file 1: Table 1.** Lines of treatment and outcomes among the studied patients’ groups. **Table 2.** Univariate and multivariate logistic regression analysis for factors associated with severe COVID-19 infection. **Table 3.** Agreement between WHO classification and COVID-19 severity index.

**Additional file 2. Figure 1.** The relation between COVID 19 severity with chest CT severity score (1A), and COVID 19 severity index (1B).

## Data Availability

All data generated or analyzed during this study are included in this published article.

## Abbreviations

ICU: Intensive Care unit
COVID-19: Coronavirus Disease 2019
SARS-CoV-2: Severe acute respiratory syndrome coronavirus 2
WHO: World Health Organization
RT-PCR: Real time reverse transcriptase-polymerase chain reaction
MIS-C: Multi-system inflammatory syndrome in children
ALC: Absolute lymphocyte count
CRP: C- reactive protein
ESR: Erythrocyte sedimentation rate
LDH: Lactate dehydrogenase
CXR: Chest X-ray
CT: Computed tomography
RSNA: Radiological society of North America
CT-SS: CT severity score
SPSS: Statistical Package for the Social Science
