# Supplementary material for: Major determinant factors of pediatric COVID-19 severity; a single center study
Source: Egypt Pediatric Association Gaz. 2023 Apr 7;71(1):22. doi: 10.1186/s43054-023-00161-2 (PMC10079495; doi:10.1186/s43054-023-00161-2)
Supplement: Supplementary file 1 — Additional file 1: Table 1. Lines of treatment and outcomes among the studied patients’ groups. Table 2. Univariate and multivariate logistic regression analysis for factors associated with severe COVID-19 infection. Table 3. Agreement between WHO classification and COVID-19 severity index. [file 43054_2023_161_MOESM1_ESM.doc]

Supplementary Table 1: Lines of treatment and outcomes among the studied patients’ groups:

| **Variables** | | **Non severe**  **No. = 34** | **Severe**  **No. = 46** | **P-value** |
| --- | --- | --- | --- | --- |
| Antibiotics | 34 (100.0%) | | 46 (100.0%) | – |
| Antiviral drugs | 15 (44.1%) | | 18 (39.1%) | 0.654 |
| Steroids  Anticoagulant therapy | 26 (76.5%)  17(50%) | | 39 (84.8%)  38(82.6%) | 0.346  0.054 |
| Intravenous immunoglobulins | 10 (29.4%) | | 33 (71.7%) | 0.001** |
| Intensive care unit admission | 0 (0.0%) | | 42 (91.3%) | 0.001** |
| Mechanical ventilation | 0 (0.0%) | | 26 (56.5%) | 0.001** |
| The need for supplemental oxygen | 6 (17.6%) | | 29 (63.0%) | 0.001** |
| Mortality  Hospital discharge | 0 (0.0%)  34 (100.0%) | | 12 (26.1%)  34(73.9%) | 0.001**  0.08 |

P**; Highly significant

Supplementary Table 2: Univariate and multivariate logistic regression analysis for factors associated with severe COVID-19 infection:

| **Variables** | **P-value** | **Odds ratio  (OR)** | **95% C.I. for OR** | | **P-value** | **Odds ratio  (OR)** | **95% C.I. for OR** | |
| --- | --- | --- | --- | --- | --- | --- | --- | --- |
|  |  |  | **Lower** | **Upper** |  |  | **Lower** | Upper |
| Lower respiratory tract symptoms | 0.024* | 2.895 | 1.150 | 7.290 | – | – | – | – |
| Fever | 0.041* | 2.266 | 1.107 | 4.194 | – | – | – | – |
| Wheezes | 0.016* | 3.187 | 1.243 | 8.173 | – | – | – | – |
| Respiratory distress | 0.024* | 2.895 | 1.150 | 7.290 | – | – | – | – |
| Neurological symptoms | 0.016* | 13.000 | 1.607 | 105.146 | – | – | – | – |
| MIS-C | 0.007** | 8.533 | 1.807 | 40.288 | – | – | – | – |
| Consolidation | 0.002** | 7.949 | 2.122 | 29.776 | – | – | – | – |
| Frequency of lobe involvement >3 | 0.023* | 3.240 | 1.176 | 8.929 | – | – | – | – |
| Clinical manifestations | 0.001** | 12.667 | 3.902 | 41.121 | <0.001** | 31.359 | 5.251 | 187.277 |
| CXR score >3 | 0.019* | 3.250 | 1.219 | 8.666 | – | – | – | – |
| Chest CT score >160 | 0.015* | 3.536 | 1.284 | 9.735 | – | – | – | – |
| Chest CT severity score (CT-SS) >9 | 0.005** | 3.947 | 1.509 | 10.327 | – | – | – | – |
| COVID-19 Severity Index >7 | 0.001** | 30.833 | 8.638 | 110.060 | – | – | – | – |
| Neutrophil/lymphocyte ratio >1.54 | 0.016* | 3.187 | 1.243 | 8.173 | – | – | – | – |
| Partial thromboplastin time (sec) > 35.4 | 0.017* | 13.588 | 1.591 | 116.032 | 0.012* | 18.763 | 0.696 | 505.502 |
| Serum ferritin (ng/ml) >537 | 0.001** | 6.500 | 2.114 | 19.987 | – | – | – | – |
| Intensive care unit admission | 0.001** | 108.500 | 22.635 | 520.082 | <0.001** | 291.456 | 17.584 | 4831.033 |
| Mechanical ventilation | 0.001** | 20.800 | 4.446 | 97.307 | – | – | – | – |
| Mortality | 0.022* | 11.647 | 1.433 | 94.681 | – | – | – | – |

MIC-C: multi-system inflammatory syndrome in children, P*; Significant, P**; Highly significant

Supplementary Table 3: Agreement between WHO classification and COVID-19 severity index

|  | **WHO classification** | | | | | |
| --- | --- | --- | --- | --- | --- | --- |
| **COVID-19 severity index** | Mild | Moderate | Severe | Critical | Total | |
| Low clinical risk | 2 | 6 | 0 | 0 | 8 (10.0%) | |
| Moderate clinical risk | 2 | 11 | 3 | 0 | 16 (20.0%) | |
| High clinical risk | 3 | 6 | 4 | 2 | 15(18.75%) | |
| Very high clinical risk | 0 | 4 | 11 | 26 | 41(51.25%) | |
| Total | 6 (7.5%) | 27 (33.75%) | 18 (22.5%) | 28 (35.0%) | | 80 |
| **Agreement statistics** | | | | | | |
| Weighted Kappa | 0.46 | | | | | |
| Standard error | 0.07 | | | | | |
| 95% CI | 0.17 to 0.48 | | | | | |
