# Supplementary figures and images for: Major determinant factors of pediatric COVID-19 severity; a single center study
Source: Egypt Pediatric Association Gaz. 2023 Apr 7;71(1):22. doi: 10.1186/s43054-023-00161-2 (PMC10079495; doi:10.1186/s43054-023-00161-2)

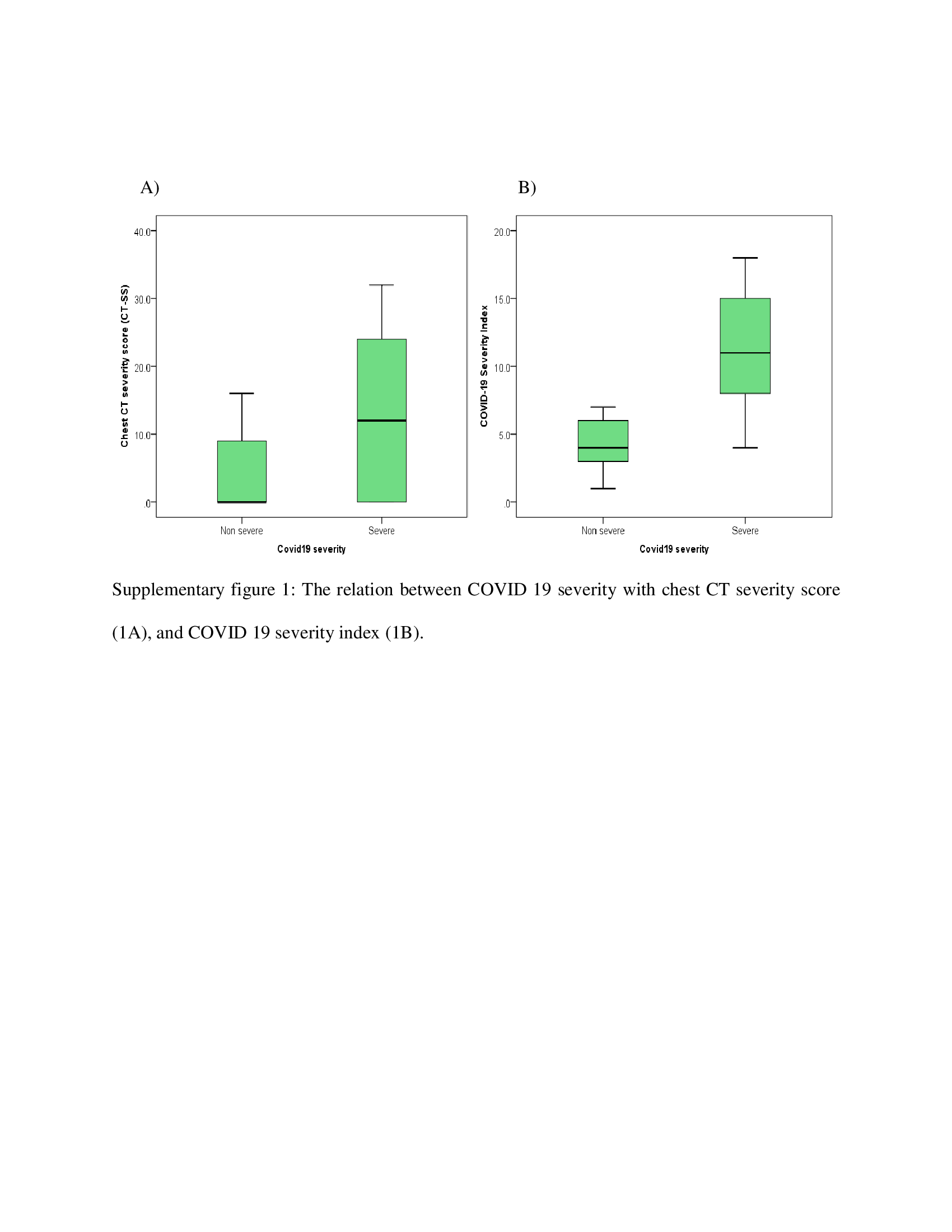

Supplement: Supplementary file 2 — Additional file 2. Figure 1. The relation between COVID 19 severity with chest CT severity score (1A), and COVID 19 severity index (1B). [file 43054_2023_161_MOESM2_ESM.tif]
